# Role of astrocytes in rhythmic motor activity

**DOI:** 10.14814/phy2.15029

**Published:** 2021-09-23

**Authors:** Alexia Montalant, Eva M. M. Carlsen, Jean‐François Perrier

**Affiliations:** ^1^ Department of Neuroscience Faculty of Health and Medical Sciences University of Copenhagen Copenhagen Denmark

## Abstract

Rhythmic motor activities such as breathing, locomotion, tremor, or mastication are organized by groups of interconnected neurons. Most synapses in the central nervous system are in close apposition with processes belonging to astrocytes. Neurotransmitters released from neurons bind to receptors expressed by astrocytes, activating a signaling pathway that leads to an increase in calcium concentration and the release of gliotransmitters that eventually modulate synaptic transmission. It is therefore likely that the activation of astrocytes impacts motor control. Here we review recent studies demonstrating that astrocytes inhibit, modulate, or trigger motor rhythmic behaviors.

## Graphical abstract

Astrocytes modulate synaptic transmission in neuronal networks that control rhythmic motor behavior. Astrocytes modulate respiration, locomotion, motor tremor, mastication and trigger switch in behaviors.

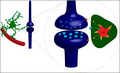

## 1 INTRODUCTION

**FIGURE 1:**
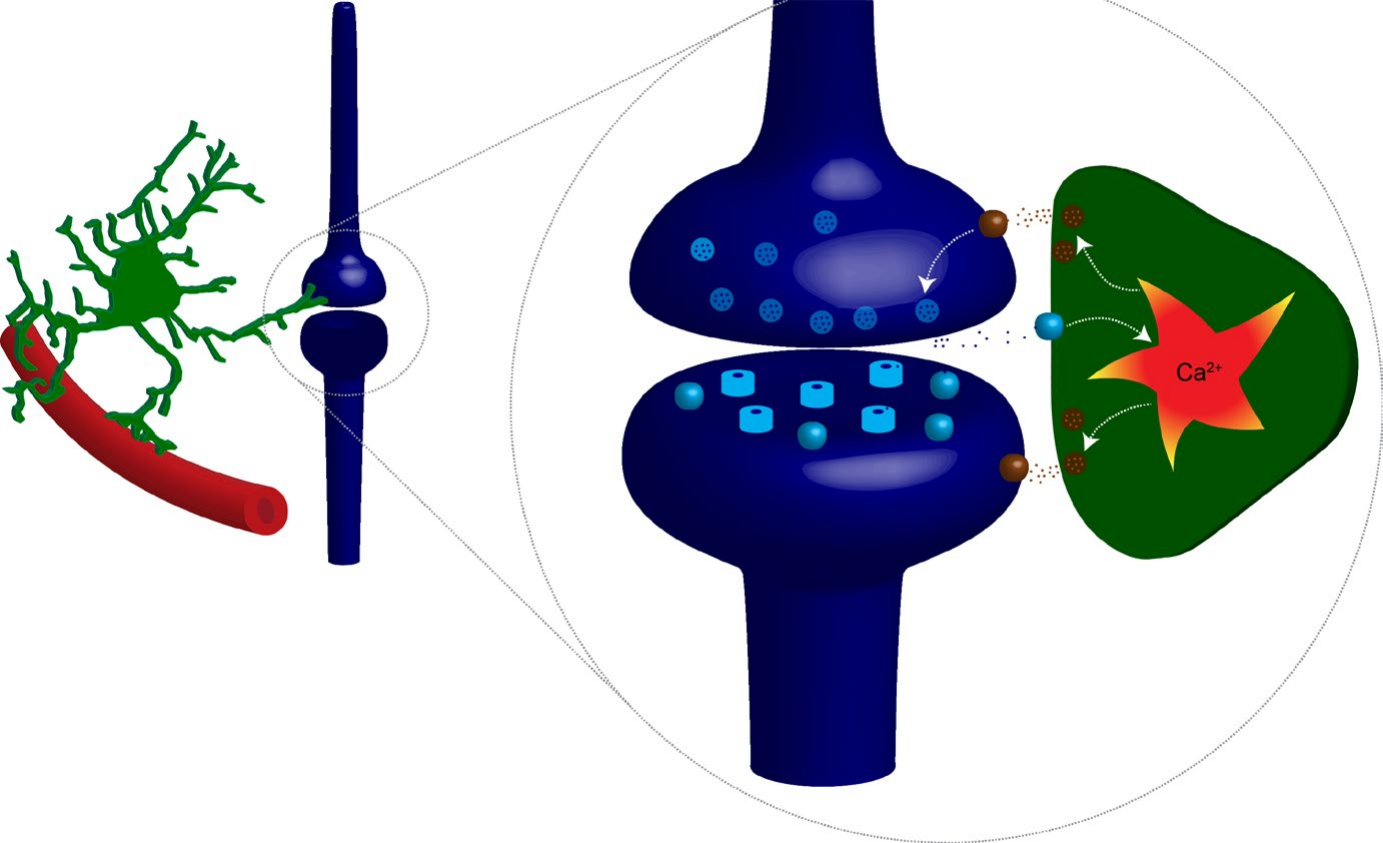
Schematic representation of the tripartite synapse. Left: astrocytes (green) are in close contact with blood vessels and synapses. Inset: in addition to postsynaptic receptors, neurotransmitters activate receptors expressed by astrocyte. This induces an increase in intracellular Ca^2+^ concentration, which triggers the release of gliotransmitters that interact with pre‐ and postsynaptic receptors

Rhythmic activity characterizes motor behaviors such as walking, chewing, or breathing. The coordinated contraction of muscles is organized by neural networks called central pattern generators (CPG) that orchestrate the timing and the activation of motoneurons in the brainstem and in the spinal cord. Classical models consider that CPGs solely consists of interconnected neurons and that the intrinsic properties of individual neurons and their synaptic connections are sufficient to explain their rhythmicity (Goulding, 2009; Harris‐Warrick, 2011; Kiehn, 2006; Marder & Bucher, 2001). However, this view is challenged by the discovery that astrocytes—the most abundant glial cell in the central nervous system—integrate and process synaptic transmission and eventually modulate neuronal activity. Astrocytes are in close contact with blood vessels and synapses (Figure 1). In contrast to neurons, astrocytes occupy non‐overlapping territories and can be chemically coupled via gap junctions. Like neurons, astrocytes respond to various neurotransmitters released from pre‐ or postsynaptic terminals, including glutamate, ATP, GABA, noradrenaline, dopamine, and endocannabinoids. This results in an increase in intracellular Ca^2+^ concentration that triggers the release of gliotransmitter molecules that in turn interact with pre‐ or postsynaptic elements and modulate synaptic transmission (Perea et al., 2009) (Figure 1). Such bidirectional interaction between neurons and astrocytes is possible because astrocytes have a complex spongiform shape with branches, branchlet, and leaflets that are in very close contact with synapses (Khakh & Sofroniew, 2015; Santello et al., 2019). Remarkably, the increase of Ca^2+^ in a given branchlet can remain spatially restricted and trigger gliotransmitter release that remains local (Grosche et al., 1999). Thus, in contrast to neurons, information processing in astrocytes does not necessarily require somatic integration of signals. Gliotransmitters released from astrocytes include ATP, glutamate, D‐Serine, and GABA (Araque et al., 2014; Carlsen et al., 2021; Christensen et al., 2013, 2018; Khakh & Sofroniew, 2015; Perea et al., 2009; Santello et al., 2019). Consequently, astrocytes have all the properties necessary for participating in information processing in the central nervous system and the number of studies demonstrating their importance in physiological functions is continuously growing. Here we will review the most recent articles demonstrating a role for astrocytes in the modulation and the genesis of motor rhythmic behaviors illustrated by breathing, locomotion, tremor, and mastication (Broadhead & Miles, 2020; Carlsen et al., 2021; Carlsen & Perrier, 2014; Christensen et al., 2013; Gourine et al., 2010; Morquette et al., 2015; Mu et al., 2019).

## 2 ROLE OF ASTROCYTES IN BREATHING

**FIGURE 2:**
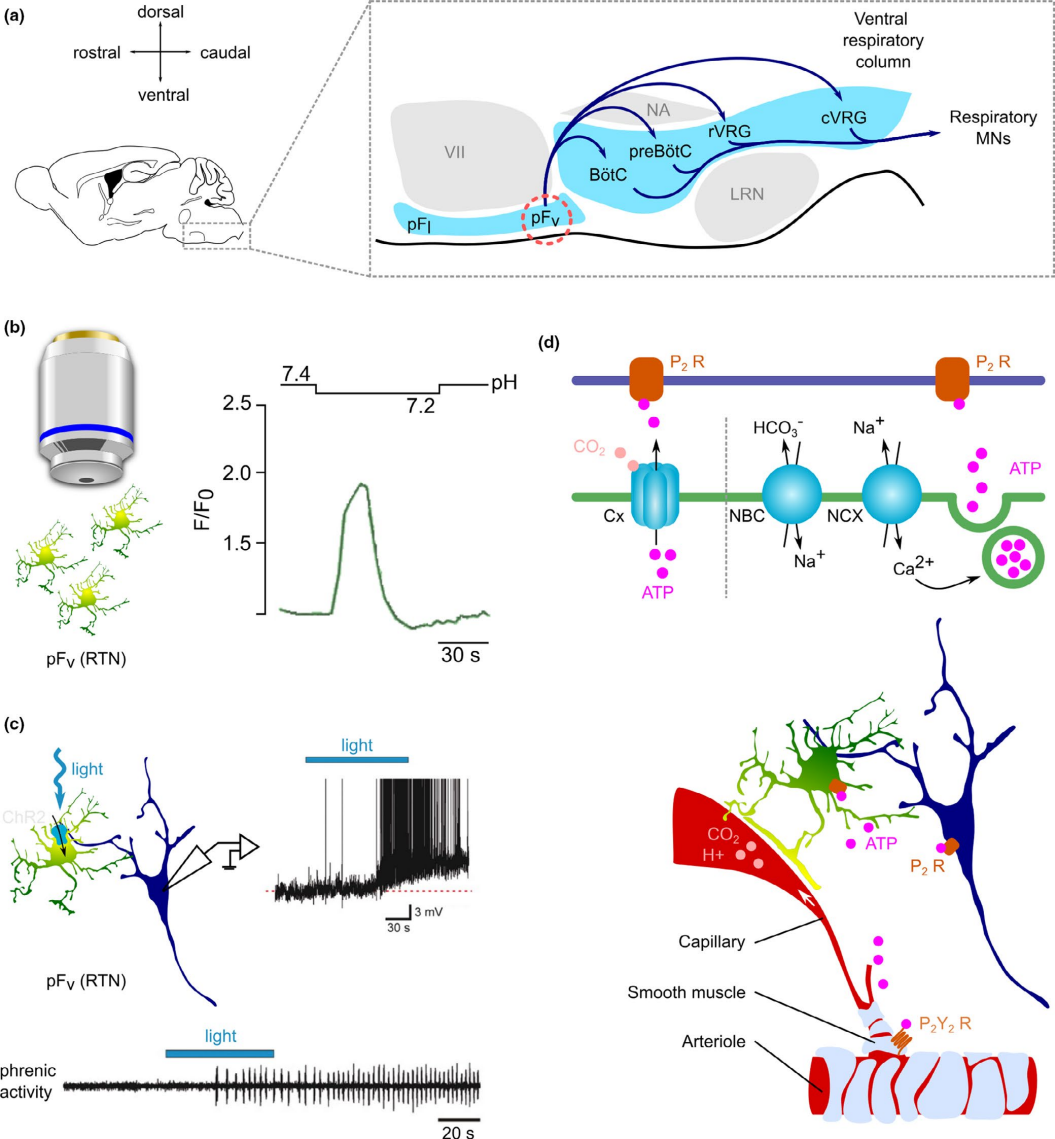
Astrocytes contribute to the modulation of breathing in response to changes in pH and pCO_2_. (a) Anatomical organization of the brainstem circuits responsible for breathing. pF_v_: ventral parafacial nucleus, also known as RTN: retrotrapezoid nucleus. preBötC: preBötzinger complex. pF: parafacial nucleus. pF_l_: lateral parafacial nucleus. pF_v_: ventral parafacial nucleus. LRN: lateral reticular nucleus. NA: nucleus ambiguus. preBötC: preBötzinger complex. BötC: Bötzinger complex. rVRG: rostral ventral respiratory group. cVRG: caudal ventral respiratory group. (b) Ca^2+^ response of astrocytes in the pF_v_ (RTN) to acidification. (c) Response of neurons from the pF_v_ (RTN) and of phrenic nerve to the optogenetic activation of pF_v_ (RTN) astrocytes. b and c are adapted from (Gourine et al., 2010), with permission. (d) Hypothetical mechanisms for the regulation of breathing by pF_v_ (RTN) astrocytes. Astrocytes sense CO_2_ increase via CO_2_‐sensitive connexin (Cx) hemichannels which directly release ATP. Acidification activates the Na^+^/HCO_3_^−^ co‐transport (NBC), which in turn activates the Na^+^/Ca^2+^ exchange (NCX). The increase in intracellular Ca^2+^ leads to the Ca^2+^‐dependent vesicular release of ATP. ATP is likely to act on several downstream targets, including neighboring astrocytes, smooth muscle cells, and neurons

Breathing is a vital behavior that ensures the intake of oxygen (O_2_) and the release of the byproduct carbon dioxide (CO_2_). It is organized by a rhythm‐generator located in the preBötzinger complex (preBötC) in the brainstem which drives the rhythmic activity of motoneurons that trigger the contraction of pump inspiratory muscles (diaphragm and external intercostal muscles) and thereby produces inspiration (Del Negro et al., 2018). Respiratory activity is continuously adjusted to metabolic activity, arterial partial pressure of O_2_ (*PaO2*), and CO_2_ (*PaCO_2_*) as well as pH so that any change in these parameters produces a modulation of the respiratory rate and/or tidal volume which brings them back to normal. These homeostatic responses are mediated by the carotid bodies and by central chemoreceptors located in the retrotrapezoid nucleus (RTN) and the preBötC (Guyenet & Bayliss, 2015) (Figure 2a). The physiology of breathing has been studied for thousands of years (French, 1978), but the possible implication of astrocytes has only recently been considered. Recent findings suggest that astrocytes play a role in the regulation of the respiratory rhythm and amplitude in response to changes in CO_2_, pH, and O_2_.

### 2.1 CO_2_ and pH sensing

The firing frequency of RTN neurons, which project to the preBötC (Figure 2a), increases in response to *PaCO_2_* rise or pH decrease (Guyenet & Bayliss, 2015). Consequently, the respiratory rate and tidal volume increase, which brings the *PaCO_2_* and pH back to normal. Astrocytes from the RTN at the ventral surface of the *medulla oblongata* play a critical role in this process. Acidification induces an increase in astrocytic intracellular Ca^2+^ concentration (Figure 2b) (Gourine et al., 2010). Importantly, the CO_2_/pH sensitivity of ventral medullary astrocytes is not shared with astrocytes from other regions of the brainstem or from the cortex (Gourine et al., 2010; Huckstepp, Id Bihi, et al., 2010; Kasymov et al., 2013), which suggests that astrocytes within distinct circuits are not interchangeable but are instead dedicated to distinct physiological functions. A CO_2_ increase (and/or a pH decrease) may initiate the activation of ventral medullary astrocytes in three ways: (1) CO_2_ activates CO_2_‐sensitive connexin hemichannels, which then release ATP (Huckstepp et al., 2010; Huckstepp, et al., 2010); (2) acidification promotes Na^+^/HCO_3_^−^ co‐transport resulting in increased intracellular Na^+^ which triggers an influx of Ca^2+^ via the Na^+^/Ca^2+^ transporter and presumably the vesicular release of gliotransmitters (Turovsky et al., 2016); (3) CO_2_ depolarizes the membrane of astrocytes by inhibiting Kir4.1‐Kir5.1 inward rectifying potassium channels, which in turn induces the release of gliotransmitters (Sobrinho et al., 2014, 2017; Wenker et al., 2010, 2012). The activation of ventral medullary astrocytes results in the release of ATP (Gourine et al., 2010; Kasymov et al., 2013). ATP amplifies the response by activating neighboring astrocytes, which in turn release more ATP, leading eventually to the depolarization of RTN neurons via the activation of P2 receptors (Gourine et al., 2010). Because RTN neurons boost the excitability of the preBötzinger complex, the activation of RTN astrocytes is expected to promote breathing. Indeed, the optogenetic activation of ventral medullary astrocytes expressing the light‐gated cation channel channelrhodopsin two triggers respiratory activity from hypocapnic apnea in anesthetized rats (Gourine et al., 2010) (Figure 2c,d). ATP, most likely released by astrocytes also acts on arterial smooth muscle cells in the RTN, counteracting the vasodilating effect of CO_2_ and maintaining arteriole tone during hypercapnia (Cleary et al., 2020; Hawkins et al., 2017). This mechanism ensures that local levels of CO_2_ remain high in the RTN and in this way maintains the drive to breathe.

### 2.2 O_2_ sensing

Because astrocytes in the preBötC respond to a decrease in *PaO_2_* by an increase in intracellular Ca^2+^ followed by the release of ATP (Angelova et al., 2015), it has been suggested that they also contribute to the homeostatic response to hypoxia (Gourine & Funk, 2017). Indeed, the disruption of purinergic signaling or of the vesicular machinery in preBötC astrocytes impairs the ventilatory response to hypoxia (Angelova et al., 2015; Rajani et al., 2018; SheikhBahaei, 2020; Sheikhbahaei et al., 2018). Once released, ATP activates metabotropic P2Y_1_ receptors expressed by preBötC neurons and stimulate breathing (Lorier et al., 2007; Rajani et al., 2018). In addition, astrocytes in the RTN sense decreases in *PaO_2_* and modulate breathing by inhibiting the internalization of TRPA1 channels, thereby promoting their accumulation at the plasma membrane, the influx of Ca^2+^ through TRPA1 channels and the subsequent release of ATP which potentiates the activity of respiratory centers (Uchiyama et al., 2020).

## 3 ROLE OF ASTROCYTES IN LOCOMOTION

Locomotion is characterized by the coordinated activation of the left and right sides of the body and by the alternation of flexor and extensor muscle contraction within the limbs. This rhythmic activity is orchestrated by CPGs located in the ventro‐medial part of the spinal cord (Kjaerulff & Kiehn, 1996). Locomotion is a flexible behavior that is continuously adjusted to external and internal conditions. For example, the most widely used psychostimulant, caffeine, promotes locomotor activity in humans and rodents (Burke, 2008; Marin et al., 2011) and exerts its effect by inhibiting adenosine receptors (Snyder et al., 1981) in the central nervous system including in the spinal cord (Acevedo et al., 2016). Since astrocytes release ATP, which can be hydrolyzed by extracellular ectonucleotidases to produce adenosine (Dunwiddie et al., 1997), they can potentially modulate locomotion. Recent evidence indeed suggests that purines released by spinal astrocytes modulate the locomotor rhythm (Witts et al., 2012). Locomotion is commonly studied with isolated spinal cord preparations from neonatal rodents in which locomotor‐like activity induced by bath application of serotonin and NMDA can be monitored by recording the electrical activity of motoneuron axons. In this model, locomotor‐like activity occurs concurrently with an increase in the frequency of Ca^2+^ events in spinal astrocytes (Broadhead & Miles, 2020). However, the astrocytic Ca^2+^ activity is neither rhythmic nor phase‐locked to the locomotor‐like rhythm. The activation of spinal astrocytes by means of chemogenetics decreases the frequency of locomotor activity by approximately 10% (Acton & Miles, 2015; Broadhead & Miles, 2020). Conversely, the poisoning of astrocytes with the gliotoxin fluoroacetate increases the frequency of locomotor activity by 20%–30% (Broadhead & Miles, 2020; Witts et al., 2012). These observations suggest that one function of spinal astrocytes could be to slow down locomotion. Because the effects of astrocyte activation are blocked by an antagonist for adenosine A1 receptors and by ectonucleotidase blockers (Acton & Miles, 2015; Broadhead & Miles, 2020; Witts et al., 2012), it is likely that astrocytes release ATP, which then gets converted into adenosine. Evidence suggests that adenosine binds to presynaptic receptors and thereby inhibits neurotransmitter release (Acton & Miles, 2015; Carlsen & Perrier, 2014).

**FIGURE 3:**
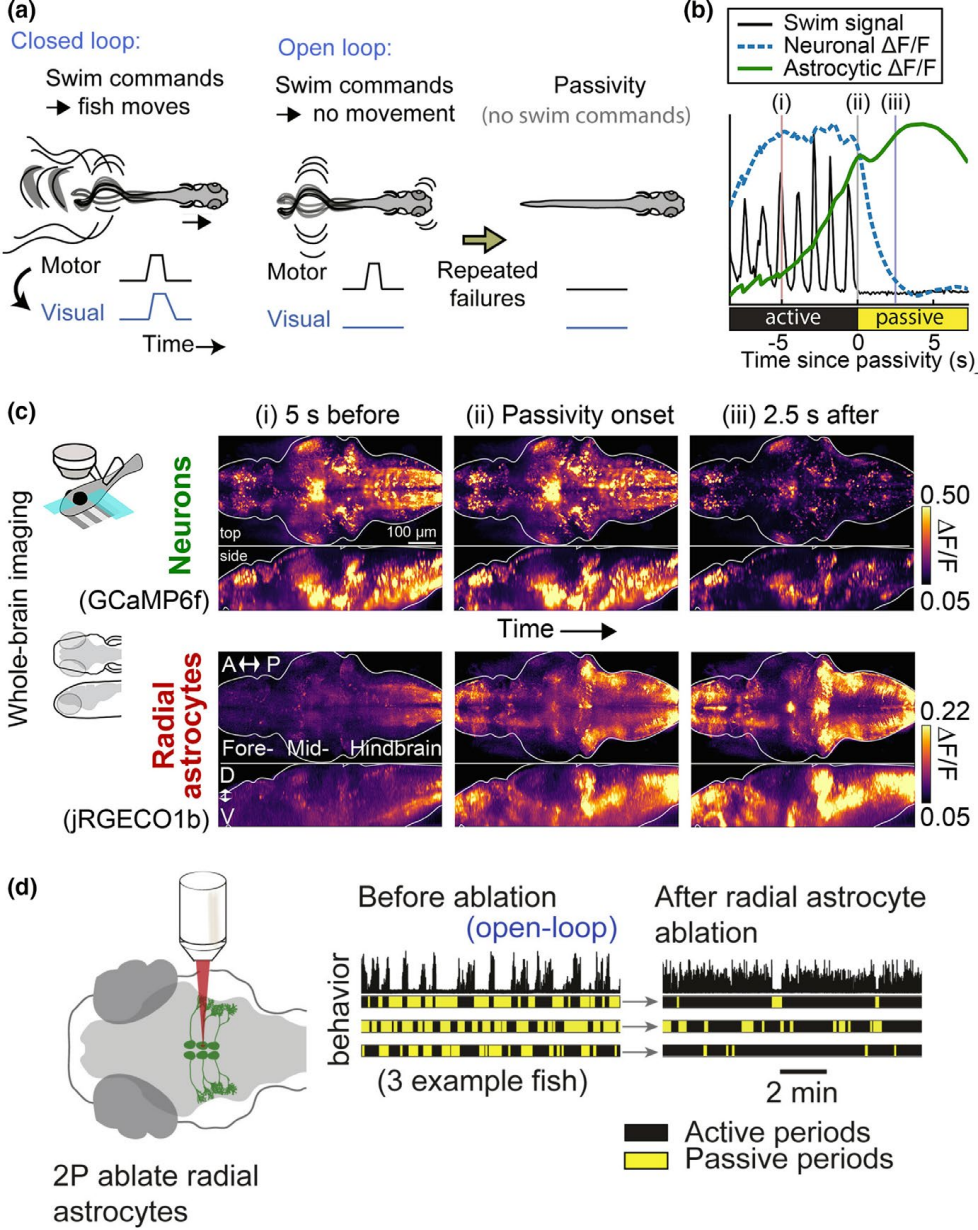
Astrocytes mediate futility‐induced passivity in larval zebrafish. (a) In closed loop, the visual feedback is adjusted to the motor activity. In open loop, the visual feedback does not match motor activity. After repeated motor failures, the animals enter a passive motor state. (b) Neuronal and glial Ca^2+^ aligned to passivity onset. (c) Whole‐brain neuronal and glial Ca^2+^ before, during, and after passivity onset. (d) The ablation of astrocytes in the lateral *medulla oblongata* reduces futility‐induced passivity. Adapted from (Mu et al., 2019), with permission

A recent study performed on larval zebrafish sheds further light on the possible role of astrocytes during locomotion (Mu et al., 2019). Paralyzed animals put in a virtual‐reality environment receive a visual feedback adjusted to their motor activity, as if they were swimming freely. Withholding visual feedback (causing all swimming attempts to seem “futile” to the animal) induces passivity within a few tens of seconds. Whole‐brain imaging shows that astrocytic Ca^2+^ in the lateral medulla oblongata rises prior to passivity onset and peaks at passivity onset (Figure 3b,c). Two strong arguments demonstrate that the activation of astrocytes triggers the behavioral state switch. First, LASER ablation of astrocytes prevents futility‐induced passivity (Figure 3d). Second, the selective activation of astrocytes by means of chemogenetics is sufficient to trigger passivity. This elegant study suggests that the computation performed by brainstem astrocytes is essential for implementing a behavioral state switch after integrating sensory information (Mu et al., 2019).

## 4 ROLE OF ASTROCYTES IN TREMOR REGULATION

Tremor is an involuntary rhythmic muscle contraction of low amplitude that occurs in all individuals. Under pathological conditions such as multiple sclerosis, stroke, or brain injury, tremor can become disabling. In a recent study, it was shown that astrocytes located in the ventral horn of the spinal cord are important for decreasing the amplitude of tremor (Carlsen et al., 2021). Active spinal interneurons from the ventral horn release endocannabinoids that bind to cannabinoid receptors type 1 (CB1) expressed by neighboring astrocytes. The resulting intracellular Ca^2+^ increase induces the release of purines that bind to presynaptic receptors and inhibits the release of excitatory neurotransmitters (Carlsen et al., 2021; Carlsen & Perrier, 2014). In animal model of essential tremor, the intrathecal injection of an agonist for CB1 receptors decreases the amplitude of tremor. This effect is suppressed when the expression of CB1 receptors is knocked out in astrocytes (Carlsen et al., 2021). These results provide a cellular mechanism for the anti‐tremor effect of cannabis reported by patients (Clifford, 1983; Consroe et al., 1997).

## 5 ROLE OF ASTROCYTES IN MASTICATION

**FIGURE 4:**
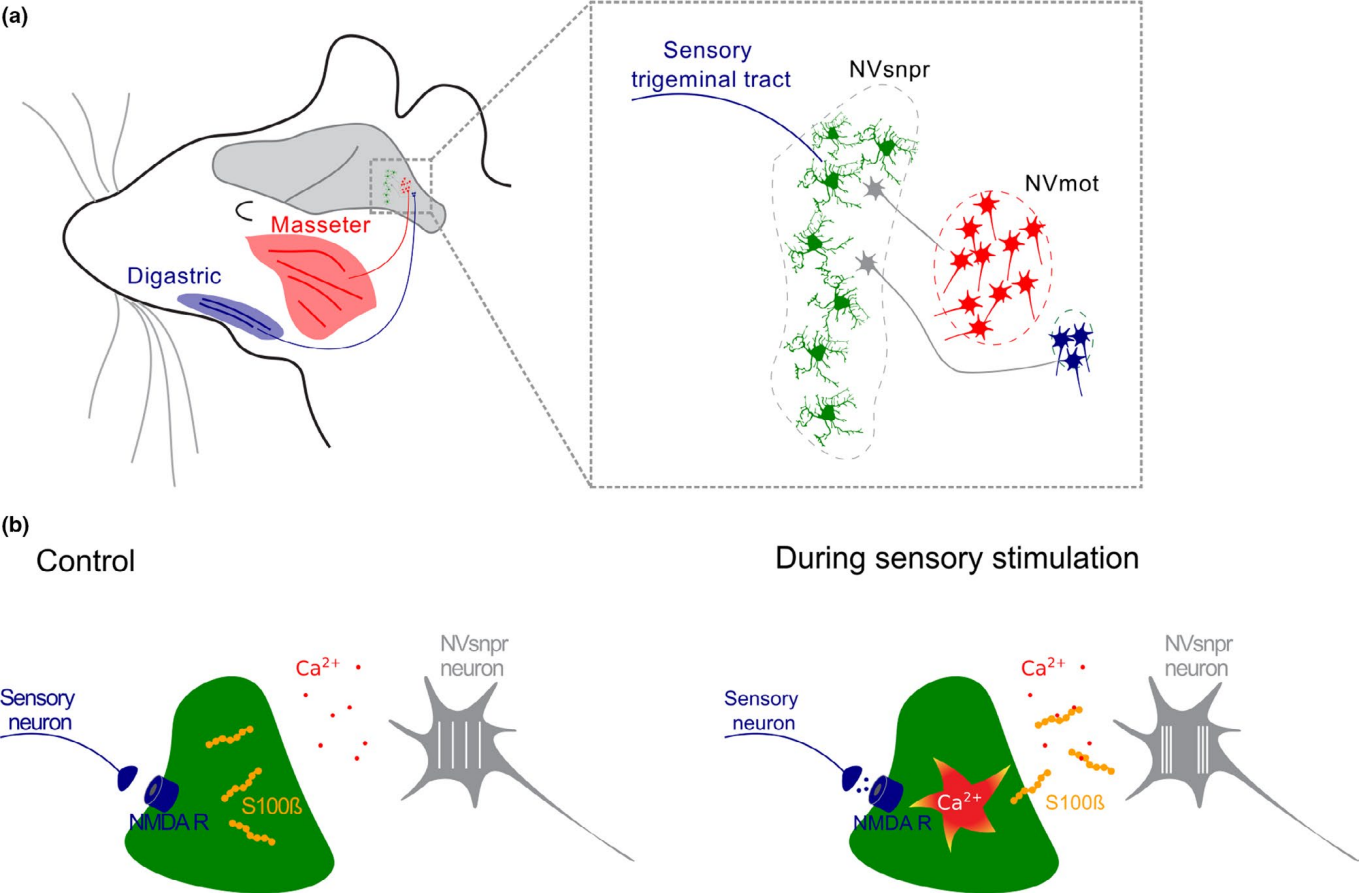
Astrocytes trigger burst firing in the masticatory CPG. (a) Anatomical organization of mastication. The main jaw opener (digastric muscle) and closer (masseter) are innervated by motoneurons from NVmot which receive inputs from NVsnpr. (b) Schematic representation of the cellular mechanism. Glutamate released from sensory trigeminal neurons activates astrocytic NMDA receptors in NVsnpr. This induces the secretion of S100ß via a Ca^2+^‐dependent mechanism. The resulting decrease in extracellular free Ca^2+^ promotes *I_NaP_* and burst firing in neighbor neurons. NVsnpr: trigeminal main sensory nucleus. NVmot: trigeminal main motor nucleus. For details, see Morquette et al. (2015)

Mastication is another example of rhythmic motor activity organized by a CPG. It is characterized by the alternated contraction of jaw opener (e.g., digastric) and jaw closer (e.g., masseter) muscles to achieve mechanical break of the food and mixing with saliva (Lund & Kolta, 2006). Both digastric and masseter muscles are innervated by trigeminal motoneurons which somata are in the trigeminal motor nucleus (NVmot) in the brainstem (Figure 4a). Sensory information originating from masticatory muscles reaches the brainstem via trigeminal primary afferents, which project to sensory nuclei including the trigeminal principal nucleus (NVsnpr) (Figure 4a). Principal cells from NVsnpr project to the thalamus as well as to diverse brainstem motor nuclei including NVmot (Morquette et al., 2012). Neurons from the dorsal part NVsnpr are characterized by persistent sodium inward currents (*I_NaP_*) and calcium‐ or sodium‐activated potassium currents (*I_K(Ca)_*, *I_K(Na)_*) (Kadala et al., 2015). The interplay between these outward and inward currents produces oscillations of the membrane potential and consequently the rhythmic firing of action potentials (Kadala et al., 2015; Morquette et al., 2012). Importantly, the duration and the amplitude of the oscillations are inversely correlated to the extracellular Ca^2+^ concentration (Brocard et al., 2006). Astrocytes from the dorsal part of NVsnpr play a critical role in the induction of the rhythmic behavior (Morquette et al., 2015). Indeed, the electrical stimulation of axons from the sensory trigeminal tract triggers the bursting behavior of neurons concomitant with a depolarization of astrocytes. Similar responses induced by local application of NMDA are abolished by buffering Ca^2+^ in astrocytes with BAPTA, suggesting that the bursting behavior induced by NMDA requires astrocytic Ca^2+^. Neuronal oscillations are triggered by astrocytes that secrete the protein S100ß that binds extracellular Ca^2+^. This promotes *I_NaP_* and the burst firing of neurons from NVsnpr (Figure 4b). One important characteristic, necessary for the masticatory behavior, is the synchronization of rhythmically active neurons. The induction of neuronal oscillations by NMDA or by stimulation of the trigeminal tract promotes coupling between astrocytes of the NVsnpr. Conversely, the selective blockade of connexin 43 abolishes NMDA‐induced rhythmic firing of NVsnpr neurons (Condamine et al., 2018). Because connexin 43 is the main protein forming gap junctions between astrocytes (but not between neurons), these results suggest that astrocytic coupling is necessary for the induction of bursting in neurons and for their synchronization.

## 6 CONCLUSION

It is now well‐established that astrocytes participate in fast information processing performed by neuronal circuits in the central nervous system (CNS). In this mini‐review, we listed some of the arguments for a role of astrocytes in different aspects of motor control. Astrocytes either process sensory inputs directly (Gourine et al., 2010) or integrate local neuronal circuit activity (Broadhead & Miles, 2020; Carlsen et al., 2021) as well as long range neuronal inputs (Morquette et al., 2015; Mu et al., 2019) and adjust motor behavior accordingly. Finally, the diversity of physiological functions of astrocytes in motor control and the variety of molecular mechanisms at play illustrates one of the fundamental concepts that have emerged in the field of neuron‐glia signaling research: neuron‐glia interactions are highly heterogeneous, with particularities related to the synaptic circuit and region involved (Araque et al., 2014; Khakh & Sofroniew, 2015). The same astrocyte can also release different gliotransmitters and exert distinct downstream effects depending on its activation pattern (Covelo & Araque, 2018). In conclusion, astrocytes are dedicated to specific physiological functions and are not interchangeable (Khakh & Sofroniew, 2015).

## CONFLICT OF INTEREST

No conflict of interest, financial, or otherwise, are declared by the authors.

## AUTHOR CONTRIBUTIONS

AM, EMMC, and JFP wrote the manuscript.
